# Coincidence of plasma cell leukemia and COVID-19: a diagnostic pitfall

**DOI:** 10.1007/s12308-023-00542-x

**Published:** 2023-04-10

**Authors:** Margot Egger, Anne Black, Christoph Robier

**Affiliations:** 1 Department of Laboratory Medicine, Konventhospital Barmherzige Brueder Linz and Ordensklinikum Linz, Seilerstaette 4, 4020 Linz, Austria; 2 Medical Faculty, Johannes Kepler University Linz, Linz, Austria; 3 Institute of Laboratory Diagnostics, Hospital of the Brothers of St. John of God, Graz, Austria; 4 Clinical Institute of Medical and Chemical Laboratory Diagnostics, Medical University of Graz, Graz, Austria

**Keywords:** Lymphocyte morphology, Plasma cell leukemia, COVID-19

## Abstract

We report the case of a 66-year-old man with a known history of IgD multiple myeloma (MM) which was admitted to hospital because of acute renal failure. Routine PCR testing on admission yielded a positive result for SARS-CoV-2 infection. Examination of the peripheral blood (PB) smear revealed 17% lymphoplasmacytoid cells and a few small plasma cells mimicking morphological changes frequently seen in viral diseases. However, flow cytometric examination showed 20% clonal lambda-restricted plasma cells being consistent with a diagnosis of secondary plasma cell leukemia. Circulating plasma cells as well as similar appearing lymphocyte subtypes such as plasmacytoid lymphocytes are frequently observed in infectious disorders such as COVID-19, so that the lymphocyte morphology in our patient’s case could have been easily misinterpreted as typical COVID-19-induced changes. Our observation highlights the importance of incorporating clinical, morphological, and flow-cytometric data in distinguishing between reactive and neoplastic lymphocyte changes because misinterpretation may affect disease classification and, beyond that, clinical decision-making, which may have serious consequences for patients.

A 66-year-old man with a known history of IgD multiple myeloma (MM) was admitted to hospital because of acute renal failure. Routine PCR testing on admission yielded a positive result for SARS-CoV-2 infection. Initial laboratory evaluation showed a plasma creatinine level of 7.23 mg/dL, leukocytosis with a white blood cell count of 14.6 × 10^9^/L, and anemia with a hemoglobin of 74 g/L. In serum protein electrophoresis, an M-spike in the gamma fraction was observed, and serum immunofixation confirmed the monoclonal IgD/lambda gammopathy. Examination of the peripheral blood (PB) smear revealed 17% lymphoplasmacytoid cells and a few small plasma cells mimicking morphological changes frequently seen in viral diseases (Fig. 1). Flow cytometric examination showed 20% clonal lambda-restricted plasma cells being consistent with a diagnosis of secondary plasma cell leukemia.

**Fig. 1:**
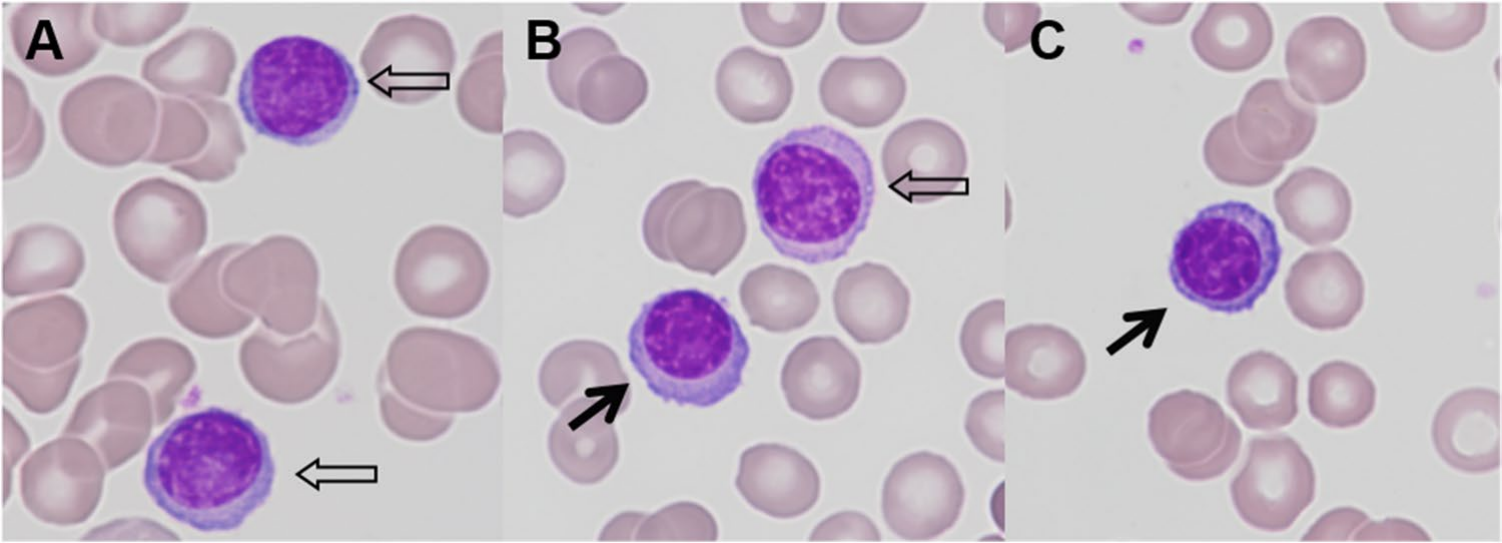
Peripheral blood smear showing **A**, **B** plasmacytoid lymphocytes (open arrow) and **B**, **C** plasma cells (filled arrow)

Circulating plasma cells as well as similar appearing lymphocyte subtypes such as plasmacytoid lymphocytes are frequently observed in infectious disorders such as COVID-19 [1, 2]. In a recently published literature review on the PB cell morphology in patients with COVID-19, it has been stated that almost all authors of previous studies on that topic, as in the current case, observed lymphoplasmacytoid cells with eccentric nuclei, plentiful and sometimes deep blue cytoplasm, and a perinuclear halo [2]. In light of these data, the lymphocyte morphology in our patient’s case could have been easily misinterpreted as typical COVID-19-induced changes. Our observation highlights the importance of incorporating clinical, morphological, and flow-cytometric data in distinguishing between reactive and neoplastic lymphocyte changes because misinterpretation may affect disease classification and, beyond that, clinical decision-making, which may have serious consequences for patients.
